# Semen HPV and IVF: insights from infection prevalence to embryologic outcomes

**DOI:** 10.1007/s10815-025-03513-6

**Published:** 2025-05-22

**Authors:** Jynfiaf Francis, Panagiotis Tsiartas, Julius Hreinsson, Maria Andersson, Jonas Hermansson, Periklis Gogas, Theophilos Papadimitriou, Cecilia Kärrberg, Mats Brännström, Randa Akouri

**Affiliations:** 1 Department of Obstetrics and Gynecology, Institute of Clinical Sciences, Sahlgrenska Academy, University of Gothenburg, Gothenburg, Sweden; 2 Department of Clinical Science, Intervention and Technology (CLINTEC), Karolinska Institute, Stockholm, Sweden; 3 Department of Infectious Diseases, Institute of Biomedicine, Sahlgrenska Academy, University of Gothenburg, Gothenburg, Sweden; 4 Department of Clinical Microbiology, Sahlgrenska University Hospital, Gothenburg, Sweden; 5 Department of Research and Development, SV Hospital Group, Angered Hospital, Angered, Gothenburg, Sweden; 6 Department of Economics, Democritus University of Thrace, Komotini, Greece

**Keywords:** Assisted reproductive technologies, Embryo, Human papilloma virus, Semen, Infertility, Machine learning, IVF outcomes

## Abstract

**Purpose:**

Human papillomavirus (HPV), the most common sexually transmitted infection, has been proposed as a potential factor in male infertility. This study aimed to assess the prevalence of HPV in semen samples from men undergoing in vitro fertilization (IVF) in Sweden and evaluate its association with semen parameters and embryological outcomes.

**Methods:**

This prospective cohort study was conducted at Sahlgrenska University Hospital, Gothenburg, Sweden, between January 2023 and February 2024. Men (*n* = 246) undergoing IVF provided fresh semen samples for HPV DNA testing using real-time PCR. Semen analysis followed WHO guidelines, and fertilization and embryo quality assessments were conducted according to the Istanbul Consensus. Machine learning (ML) models were employed to predict fertilization and blastocyst formation outcomes.

**Results:**

HPV was detected in 8.9% of semen samples. No significant differences in semen parameters were found between HPV-positive and HPV-negative men. However, in the non-male infertility subgroup, HPV-positive men had significantly higher total motility (median 65 vs. 60%, *p* = 0.021) and progressive motility (median 65 vs. 55%, *p* = 0.016). Similarly, in the unexplained infertility subgroup, progressive motility was higher in HPV-positive men (median 60 vs. 50%, *p* = 0.033). No significant differences were found in fertilization or blastocyst formation rates, and ML analysis confirmed that HPV presence did not influence predictive model accuracy.

**Conclusion:**

HPV is detectable in the semen of a notable number of men undergoing IVF, but its presence does not significantly impact fertilization or embryo development. These findings suggest that routine HPV screening in semen may not be necessary for predicting IVF outcomes.

**Trial registration:**

The study was registered on ClinicalTrials.gov (ID: NCT06161727).

**Supplementary Information:**

The online version contains supplementary material available at 10.1007/s10815-025-03513-6.

## Introduction

Infertility is a multifaceted condition affecting a substantial portion of the global population, defined clinically as the inability to conceive after one year of regular, unprotected intercourse [1]. According to the World Health Organization (WHO), the lifetime prevalence of infertility is estimated at 17.5% [2].

Assisted reproductive technologies (ART), including in vitro fertilization (IVF) and intracytoplasmic sperm injection (ICSI), have become crucial in addressing infertility by overcoming specific reproductive barriers and significantly enhancing conception rates [3]. ART is particularly beneficial in cases of male infertility. The cause of infertility is solely attributed to the male partner in 20–30% of cases, while a male factor contributes to an additional 20%, making male infertility responsible for nearly 50% of all infertility cases [4]. Male infertility is a growing public health concern, driven by a combination of environmental, genetic, lifestyle, and infectious factors [5–7].

Human papillomavirus (HPV) is the most prevalent sexually transmitted viral infection worldwide, with its epidemiology well-established in women [8]. However, data on HPV prevalence among men are limited [9]. Over 200 HPV genotypes are known, all with a strong tropism for mucosal membranes [10]. While HPV is best known for its role in the development of various premalignant and malignant diseases [11], at least 13 high-risk HPV (HR-HPV) genotypes are directly associated with the development of cervical cancer and other cancers [8, 12]. Sweden introduced a school-based HPV vaccination program for girls in 2012, achieving an estimated coverage of approximately 80% [13]. Since 2020, the program has been extended to include boys. Following the implementation of the HPV vaccination program, a decline in the prevalence of HPV 16 has been observed among vaccinated individuals.

Emerging evidence indicates that HPV prevalence in semen is higher among infertile men than in the general population, with prevalence ranging from 10 to 35.7% in infertile individuals compared to 2 to 31% in fertile counterparts [14, 15]. The presence of HPV in semen can negatively affect sperm motility and quality, with HR HPV particularly compromising sperm DNA integrity and impairing fertility [16–19]. However, inconsistencies in study methodologies and small sample sizes have resulted in conflicting findings [20].

HPV infection poses implications for ART, particularly ICSI, where the risk of injecting HPV-infected sperm could affect reproductive outcomes [21]. HPV has been shown to localize to the sperm head’s equatorial region, raising concerns about transmission during fertilization and its potential impact on early embryonic development [16, 22]. Despite some studies suggesting an increased risk of miscarriage associated with HPV in semen [21, 23], conclusive evidence linking HPV to compromised ART pregnancy outcomes is lacking [21, 24].

Machine learning (ML), introduced in the 1960 s, enables artificial intelligence (AI) systems to learn from data, recognize complex patterns, and make decisions with minimal human intervention [25]. In the field of IVF, ML is increasingly being used to support embryo selection and to improve clinical outcomes, as it can process large datasets to estimate the likelihood of treatment success [25–30]. Unlike traditional statistical models, ML approaches are data-driven and capable of detecting complex, non-linear patterns without relying on predefined assumptions. This makes them a promising tool for examining hypotheses, such as whether HPV infection influences embryological outcomes. However, challenges remain, including establishing regulatory frameworks to ensure the safe use of AI in research in reproductive medicine [29, 31].

The main aim of this study was to determine the point prevalence of HPV and of both HR and low-risk (LR) HPV types in semen samples from men undergoing IVF in Sweden, where data on this topic are limited. The study also examined the relationship between HPV status and semen parameters (volume, concentration, and motility) and its impact on fertilization and blastocyst formation. A secondary objective was to leverage both traditional statistical and ML models to enhance the predictive analysis of reproductive outcomes in relation to HPV prevalence. This is the first study in Sweden to incorporate ML methodologies to explore the potential influence of seminal HPV on embryological results.

## Materials and methods

### Study design

This prospective cohort study was conducted at the Division of Reproductive Medicine, Sahlgrenska University Hospital, Gothenburg, Sweden, from January 2023 to February 2024. Couples intending to undergo IVF treatment utilizing autologous gametes during this period were invited to participate through self-selection sampling. Male partners provided fresh semen samples for HPV analysis, while female partners consented to the collection of relevant clinical data from their medical records. Detailed study information was sent to participants by mail weeks before their initial hospital visit, during which further verbal explanations of the study were provided. Recruitment was conducted on an ongoing basis throughout the study period.

To ensure population homogeneity and mitigate potential selection bias, specific groups were excluded: couples undergoing preimplantation genetic testing, those using donated gametes, individuals pursuing fertility preservation, sperm donors, and couples where the male partner used surgically retrieved sperm or frozen sperm. These exclusions were justified as follows: 1. omitting preimplantation genetic testing enhanced the generalizability of results by avoiding biases linked to genetic embryo selection; 2. excluding donor gamete treatments upheld methodological consistency, given their distinct physiological and psychological implications; 3. excluding fertility preservation cycles, which follow unique protocols and medication regimens, prevented potential confounding outcome influences.

*Ethical approval.*

Ethical approval was obtained from the Swedish Ethical Review Authority (Dnr 2022–03339-01 and 2023–04431-02). The study’s aims were carefully explained to participants to ensure informed consent, with written consent obtained from all participants.

### Study population and data collection

**Fig. 1:**
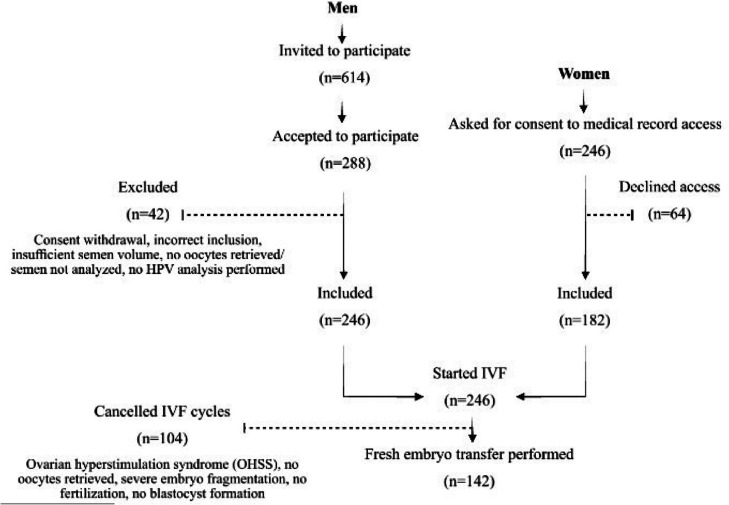
This flowchart illustrates the stepwise progression of participant inclusion and exclusion in the study

The study included male partners of couples undergoing IVF, with or without ICSI. A total of 614 men were invited to participate, and 288 accepted, resulting in a participation rate of 47%. Eligible participants provided fresh semen samples in sufficient quantities for standard semen analysis and HPV DNA testing. Forty-two men were excluded due to various reasons, including withdrawal of consent, insufficient semen volume, and absence of retrieved oocytes in their partners, which precluded sperm analysis. Consequently, 246 participants were included in the final study cohort (Fig. 1). Clinical data were sourced from the Reproductive Medicine electronic databases Melior (Oracle Corporation, Austin, TX, USA) and WinIVF (Livio, Gothenburg, Sweden).

For male participants, collected data included age (years), body mass index (BMI, kg/m^2^), health status (including prior diseases), lifestyle habits (smoking and snuff use), and paternity history. Semen parameters assessed pre- and post-preparation included semen volume (mL), total sperm count (10^6^), sperm concentration (10^6^/mL), total motility (%), progressive and non-progressive motility (%), and motility score.

For female partners, data included age (years), BMI (kg/m^2^), smoking and snuffing habits, health status (including past diseases), and parity. Infertility diagnoses, duration of infertility (months), and specific IVF treatment details were recorded, covering ovarian stimulation protocols (agonist or antagonist), total gonadotropin dosage (IU), treatment duration (days), cycle cancellations, number of retrieved oocytes, fertilization method (conventional IVF or ICSI), number of oocytes undergoing insemination or ICSI (fertilization exposure), and fertilized oocytes, blastocyst formation, and cryopreserved embryos. Demographic data were collected exclusively from consenting female partners.

The fertilization rate was defined as the proportion of fertilized oocytes, marked by the presence of two pronuclei, relative to the total number of oocytes exposed to fertilization. The blastocyst formation rate, serving as an indicator of embryo quality, was the proportion of fertilized oocytes developing into blastocysts within a 5- to 6-day culture period, calculated as the number of blastocysts formed divided by the number of fertilized oocytes. Presence or absence of blastocysts five days post-fertilization was used as a key marker of embryo quality and IVF success.

### Semen analysis and preparation for IVF

Routine semen analysis and preparation were performed according to established protocols by trained embryologists at the Laboratory of Reproductive Medicine, Sahlgrenska University Hospital, adhering to the WHO’s laboratory manual for semen analysis [32]. The laboratory follows a comprehensive quality management system, including standard operating procedures (SOPs) and internal and external quality control measures, ensuring precision and adherence to international best practices.

Semen samples were collected after 2–7 days of abstinence and allowed to liquefy for 10–30 min on a Tilt Mixer 440 (Swelab Instrument AB, Stockholm, Sweden). Ejaculate volume (mL) was measured using a pipette, and a 10 µL aliquot was examined under light microscopy for motility categorization using a 3-point scale (non-progressive, slow progressive, and fast progressive motility). Sperm concentration (10^6^/mL) was determined after mixing a 50 µL sample aliquot with 4.95 mL of 0.9% NaCl solution containing 0.5% formaldehyde, followed by counting in a Bürker™ Counting Chamber (depth 0.100 mm). Total sperm concentration (10^6^) was calculated by multiplying the concentration by sample volume. Sperm preparation utilized a two-step density gradient centrifugation. A 40% PureSperm™ (Nidacon, Gothenburg, Sweden) gradient was layered over an 80% gradient in a test tube. Up to 2 mL of semen was layered over the 40% gradient and centrifuged at 300 × *g* for 20 min. Post-centrifugation, non-viable sperm and debris were removed, leaving a pellet resuspended in 5 mL of G-IVF™ PLUS medium (Vitrolife, Gothenburg, Sweden) for further centrifugation at 500 × *g* for 10 min. Final sperm concentration was verified microscopically, with additional dilution performed if required. An aliquot (10 µL) of the prepared sample was analyzed microscopically to re-assess motility and sperm parameters.

### Ovarian stimulation and embryo culture

Ovarian stimulation was individualized based on age, BMI, and antral follicle count. Stimulation protocols involved recombinant follicle-stimulating hormone or human menopausal gonadotropins, following either a gonadotropin-releasing hormone (GnRH) agonist or antagonist protocol. Final oocyte maturation was triggered with either human chorionic gonadotropin or a GnRH agonist approximately 36 h before oocyte retrieval, performed via transvaginal ultrasound-guided aspiration.

Fertilization was performed using either conventional IVF or ICSI, dependent on semen quality. ICSI was selected in cases where semen parameters were suboptimal according to the World Health Organization (WHO) criteria, and/or when the number of motile sperm after semen preparation on the day of oocyte retrieval was lower than 1 million. In all other cases, conventional IVF was applied. Male factor infertility was defined per WHO criteria (presence of ≥ 1 semen parameter abnormality) [33]. Fertilization assessment was conducted 16–18 h post-insemination or injection, based on the presence of two pronuclei. Embryos were cultured in an ES + time-lapse incubator (Vitrolife) using G-TL® culture medium (Vitrolife) until transfer assessment. Embryo transfers occurred on days 2, 3, or 5, based on development. Surplus blastocysts were vitrified. High ovarian hyperstimulation syndrome risk warranted a freeze-all protocol. Embryo quality was scored according to the Istanbul Consensus [34].

### HPV DNA extraction, detection, and genotyping

For HPV testing, 100 µL of the fresh semen was mixed with 300 µL of ThinPrep™ PreservCyt™ solution (Hologic Sweden AB, Kista, Sweden), stored at – 20 °C until analysis at the Clinical Microbiology Laboratory, Sahlgrenska University Hospital. HPV DNA extraction employed the DNA and Viral NA Small Volume Kit with the MagNA Pure 96 system (Roche Molecular, Mannheim, Germany). Real-time PCR was performed in eight multiplex reactions with Universal Mastermix (Applied Biosystems, Foster City, CA, USA) and type-specific primers and probes for 14 HPV genotypes and the beta-globin gene. HR HPV types included 16, 18, 31, 33, 35, 39, 45, 51, 52, 56, 58, and 59, while LR HPV types included 6 and 11 [35, 36]. Beta-globin Ct values > 35 indicated insufficient cellular material for reliable HPV assessment, while HPV Ct values < 40 indicated positive specimens.

### Statistical analyses

The required sample size for estimating the point prevalence of HPV positivity in sperm among men undergoing IVF was calculated using a *Z*-score of 1.96 for a 95% confidence level, an expected prevalence (*p*) of 10%, and a margin of error (*d*) of 5%. This initial calculation yielded a sample size of 139 participants. To account for a potential 15% dropout rate, the adjusted sample size was set at approximately 164 participants, ensuring sufficient statistical power for reliable HPV prevalence estimates in the target population.

Comparative analyses of HPV semen status (positive vs. negative) were conducted for both continuous and categorical variables. The Mann–Whitney *U* test was used for continuous variables due to the non-normal data distribution. Comparisons within the HPV-positive group (between HR and LR HPV subcategories) were performed using the Kruskal–Wallis test, followed by Dunn’s non-parametric multiple comparisons to control for type I error. Categorical variables were analyzed using Pearson’s chi-square test, while Fisher’s exact test was applied when the assumptions for the chi-square test were not met, ensuring robustness in smaller samples.

To explore the association between HPV semen status (with and without HR/LR differentiation) and semen parameters, linear regression models were employed. Univariate analyses were initially conducted to identify potential associations, and variables with *p*-values below 0.2 were included as potential confounders in multivariate models to account for any overlooked effects. All regression models adjusted for potential confounders such as male age, BMI, smoking status, and snuff use.

Additional comparisons of HPV-negative and HPV-positive men were performed across infertility subcategories (non-male, unexplained, and male infertility) to assess HPV’s impact within distinct clinical contexts. Linear regression models were used for continuous IVF outcomes (e.g., fertilization rate, blastocyst formation rate, number of blastocysts formed, and cryopreserved), while logistic regression was applied to the binary outcome of blastocyst formation. These models were adjusted for relevant factors such as the number of retrieved oocytes, total progressively motile sperm after preparation, duration of infertility, male and female ages, sperm concentration after preparation, and the fertilization method. All statistical analyses were conducted using SPSS (v26.0), with a significance threshold of 0.05 or less.

### Data analysis with machine learning algorithms

Four key ML algorithms were employed to enhance predictive analysis: support vector machines (SVM), decision trees (DT), random forests (RF), and extreme gradient boosting (XGBoost). Additionally, the elastic net logistic regression (Logit) was used as a relevant benchmark. The SVM algorithm, known for its ability to create an optimal hyperplane and efficiently handle non-linear data using kernel functions, was included for its robust performance in classification tasks [37]. DTs provide interpretable, tree-like structures for efficient classification and prediction, with each split within the decision tree designed to reduce overall data impurity [38]. RFs improve upon the DT algorithm by employing ensemble bootstrapping, thereby reducing overfitting and enhancing the model’s generalization ability to new and unseen data [39]. XGBoost, an advanced boosting algorithm, iteratively builds decision trees to reduce bias and optimize model performance [40]. The elastic net model, which combines ridge (L2) and lasso (L1) regression techniques, served as a benchmark due to its flexible regularization and strong generalization abilities [41].

Model training followed a two-step process. First, the dataset was split, with 80% allocated for in-sample (training) and 20% reserved for out-of-sample (validation) analysis on unseen data. The in-sample data were used to identify the best-performing model using 39 independent variables (Supplementary Tables 1 and 2). The out-of-sample data were then fed into the optimally trained ML models to formally assess their generalizability. Subsequently, a fivefold cross-validation scheme was applied during training, dividing the dataset into five equal parts. Each set of hyperparameters was tested across these folds, with one fold serving as the test set for validation and the remaining folds used for model training in each iteration. This process was repeated for each hyperparameter set, and the average accuracy across the test folds determined the in-sample model performance [42].

SVM models were trained using linear, radial basis function (RBF), and polynomial kernels. Model selection was based on accuracy, defined as the ratio of correctly predicted observations to the total number of observations. The best-performing model underwent a variable importance measure (VIM) analysis to determine the relative contribution of each variable to the outcome prediction. Variables were ranked by gain, representing the improvement in model accuracy attributable to each feature.

## Results

During the study period, a total of 246 men initiated IVF treatment and consented to participate in the study, while 182 of their female partners also provided consent to participate.

No statistically significant differences were found in clinical and semen parameters when comparing HPV-negative and HPV-positive men (Table 1). The point prevalence of HPV in semen samples was 8.9%. A trend toward higher total sperm motility was noted in HPV-positive men (median 60%) compared to HPV-negative men (median 50%). However, this difference did not reach statistical significance after adjusting for potential confounders, including male age, BMI, smoking status, and snuff use (adjusted *p* = 0.057). Other semen parameters, such as sperm concentration and progressive motility, also showed no significant differences between the groups.

**Table 1:** Clinical and semen parameters between HPV-negative and HPV-positive men

	**HPV-negative** (*n* = 224; 91%)	**HPV-positive** (*n* = 22; 8.9%)	**HR HPV-positive** (*n* = 19; 7.7%)	**LR HPV-positive** (*n* = 3; 1.2%)	Adjusted *p*1-value	Adjusted *p*2-value
Age (years); median (IQR)	34 (7)	34.5 (5)	35 (6)	33 (5)	0.72	0.59
BMI (kg/m^2^); median (IQR)	25 (4)	26 (5)	26 (5)	27 (2)	0.35	0.77
Absence of previous diseases; *n* (%)	185 (83)	18 (82)	15 (78.9)	3 (100)	0.93	0.63
Smoking status; *n* (%)	9 (4)	0 (0)	0 (0)	0 (0)	1.00	1.00
Snuffing status; *n* (%)	67 (30)	8 (36)	7 (36.8)	1 (33)	0.53	0.40
Previous children; *n* (%)	12 (5)	0 (0)	0 (0)	0 (0)	0.27	0.66
Indication to IVF; *n* (%)					0.92	0.87
Male infertility	79 (35)	8 (36)	8 (42.1)	0 (0)		
Unexplained infertility	91 (41)	12 (55)	10 (52.6)	2 (66.7)		
Disovulatory	26 (12)	2 (9)	1 (5.3)	1 (33.3)		
Endometriosis	12 (5)	-	-	-		
Tubal infertility	8 (4)	-	-	-		
Uterine infertility	4 (2)	-	-	-		
Oocyte factor	4 (2)	-	-	-		
Semen volume (mL); median (IQR)	3.2 (1.9)	2.4 (2.6)	2.4 (2.8)	2.9 (0.9)	0.12	0.12
Sperm concentration (10^6^/mL); median (IQR)	56 (66)	53 (71)	48 (62)	95 (137)	0.67	0.67
Total sperm count (10^6^); median (IQR)	121 (202)	110 (169)	91 (141)	209 (429)	0.52	0.52
Total sperm motility (%); median (IQR)	50 (20)	60 (20)	60 (20)	80 (30)	0.057	0.36
Progressive motile sperm (%); median (IQR)	50 (20)	60 (25)	60 (28)	80 (30)	0.14	0.14
Non-progressive motile sperm (%); median (IQR)	0 (0)	0 (5)	0 (5)	0 (0)	0.26	0.26
Sperm motility score; median (IQR)	2.5 (0)	2.5 (0.25)	2.5 (0.25)	2.5 (0.25)	0.18	0.18

^*HPV*^^, human papillomavirus; *HR HPV*, high−risk human papillomavirus; *LR HPV*, low−risk human papillomavirus; *BMI*, body mass index; *IQR*, interquartile range. Adjusted p1−value: statistical comparison between HPV−negative and HPV−positive groups; adjusted p2−value: statistical comparison between HR HPV−positive, LR HPV−positive, and HPV−negative groups. Adjustments were made for male age, BMI, smoking status, and snuffing status^

Table 2 shows the distribution of HR and LR HPV genotypes detected in positive semen samples. Among the HR HPV genotypes, which accounted for 86.3% of HPV-positive cases, HPV 16 was identified in one case (0.4% of the entire cohort and 4.5% of HPV-positive cases). HPV 51 was the most prevalent genotype, detected in five cases, corresponding to 2% of the total cohort and 22.7% of HPV-positive individuals. For LR HPV genotypes, which comprised 13.6% of HPV-positive cases, only HPV 6 was observed, detected in three cases (1.2% of the total cohort and 13.6% of HPV-positive cases).

**Table 2:** Distribution of high-risk HPV (HR HPV) and low-risk HPV (LR HPV) genotypes. The table lists the number and percentage of semen infections for each genotype of HR HPV and LR HPV among the whole cohort of men and the 22 HPV-positive cases

HR HPV genotypes	Number of detections (*n* = 19)	Whole cohort (*n* = 19/246, 7.7%)	HPV-positive cases (*n* = 19/22, 86.3%)
HPV 16	1	0.4%	4.5%
HPV 39	1	0.4%	4.5%
HPV 45	3	1.2%	13.6%
HPV 51	5	2%	22.7%
HPV 52	3	1.2%	13.6%
HPV 56	3	1.2%	13.6%
HPV 58	3	1.2%	13.6%
**LR HPV genotypes**	**Number of detections** **(*n* = 3)**	**Whole cohort** **(*n* = 3/246)**	**HPV-positive cases** **(*n* = 3/22)**
HPV 6	3	1.2%	13.6%

Comparisons of semen parameters between HPV-negative and HPV-positive men across infertility subcategories indicated significant differences in certain sperm motility measures (Table 3). In the non-male infertility subgroup (female and unexplained infertility), HPV-positive men had significantly higher total sperm motility compared to HPV-negative men (median 65 vs. 60%, adjusted *p* = 0.021). Additionally, progressive motile sperm was significantly higher in HPV-positive men (median 65 vs. 55%, adjusted *p* = 0.016). No significant differences were noted in semen volume, sperm concentration, or total sperm count. Within the unexplained infertility subgroup, HPV-positive men showed higher progressive motility compared to HPV-negative men (median 60 vs. 50%, adjusted *p* = 0.033), with total sperm motility differences approaching significance (adjusted *p* = 0.051). Other parameters, including semen volume, sperm concentration, and total sperm count, did not show significant differences. In the male infertility subgroup, no significant differences were observed in motility scores, progressive motility, or other semen parameters between the groups, except for a higher non-progressive motility in HPV-positive men (median 2.5 vs. 0%, adjusted *p* = 0.025).

**Table 3:** Comparison of semen parameters between HPV-negative and HPV-positive men across infertility subcategories

Infertility category	Non-male infertility (Female and unexplained infertility) (*n* = 159; 65%)			Unexplained infertility (*n* = 103; 42%)			Male infertility (*n* = 87; 35%)		
	**HPV-negative** **(*n* = 145; 91%)**	**HPV-positive** **(*n* = 14; 9%)**	**Adjusted *p*-value**	**HPV-negative** **(*n* = 91; 88%)**	**HPV-positive** **(*n* = 12; 12%)**	**Adjusted *p*-value**	**HPV-negative** **(*n* = 79; 91%)**	**HPV-positive** **(*n* = 8; 9%)**	**Adjusted *p*-value**
Semen volume (mL); median (IQR)	3.2 (1.8)	2.6 (2.6)	0.54	2.9 (1.8)	2.4 (3.2)	0.64	3.2 (2.3)	2 (2.6)	0.10
Sperm concentration (10^6^/mL); median (IQR)	67 (68)	51 (65)	0.45	67 (79)	47 (54)	0.19	38 (60)	59 (102)	0.22
Total sperm count (10^6^); median (IQR)	196 (203)	160 (110)	0.73	191 (106)	152 (106)	0.12	31 (41)	23 (23)	0.33
Total sperm motility (%); median (IQR)	60 (20)	65 (16)	0.021	60 (15)	60 (18)	0.051	45 (20)	48 (28)	0.53
Progressive motile sperm (%); median (IQR)	55 (15)	65 (11)	0.016	50 (18)	60 (14)	0.033	40 (25)	38 (24)	0.89
Non-progressive motile sperm (%); median (IQR)	0 (0)	0 (0)	0.38	0 (0)	0 (0)	0.29	0 (5)	2.5 (18)	0.025
Sperm motility score; median (IQR)	2.5 (0.25)	2.6 (0.25)	0.052	2.5 (0.25)	2.5 (0.25)	0.28	2.25 (0.25)	2.4 (0.25)	0.58

Data are presented as median (IQR: interquartile range). The *p*-values indicate the statistical significance of differences between HPV-negative and HPV-positive groups within each infertility subcategory. Adjustments were made for male age, BMI, smoking status, and snuffing status

**Fig. 2:**
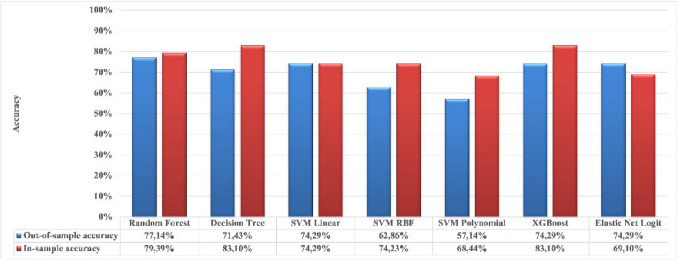
Comparison of the accuracy of different machine learning models based on in-sample and out-of-sample performance. The blue bars represent the out-of-sample accuracy, while the red bars indicate the in-sample accuracy for each model tested

Table 4 outlines the clinical parameters and IVF outcomes based on the semen HPV status. Key baseline characteristics, including infertility duration, female age, and BMI, showed no significant differences between the two groups. However, a significantly higher rate of previous pregnancies was reported among women whose partners were HPV-positive (59%) compared to those with HPV-negative partners (32%) (*p* = 0.03). IVF treatment parameters did not show significant differences based on HPV status. Outcomes such as the number of blastocysts formed and cryopreserved, fertilization rates, and blastocyst formation rates were comparable, with adjusted *p*-values indicating no significant differences. The median fertilization rate was slightly higher in the HPV-positive group (71 vs. 70%), though this did not reach statistical significance (adjusted *p* = 0.07). Blastocyst formation rates were also similar between the groups (33% for HPV-negative vs. 22% for HPV-positive participants, adjusted *p* = 0.96). Predictive modeling for blastocyst formation identified XGBoost and DT as the best-performing models. XGBoost showed superior performance, with an in-sample accuracy of 83.10% and an out-of-sample accuracy of 74.29% (Fig. 2).

**Table 4:** Comparison of clinical parameters and outcomes in couples undergoing IVF treatment: HPV-negative vs. HPV-positive men

	**HPV-negative** (*n* = 224; 91.1%)	**HPV-positive** (*n* = 22; 8.9%)	*P*-value	Adjusted *p*-value
**Female partners** (*n* = 182; 74%)	(*n* = 165; 91%)	*(n* = 17; 9%)		
Declined inclusion: (*n* = 64; 26%)				
Infertility duration (months); median (IQR)	30 (18)	36 (24)	0.36	
Age (years); median (IQR)	32 (4)	33 (6)	0.08	
BMI (kg/m^2^); median (IQR)	25 (7)	25 (9)	0.99	
Absence of previous diseases; *n* (%)	113 (69)	12 (71)	0.86	
Smoking status; *n* (%)	2 (1)	1 (6)	0.25	
Snuffing status; *n* (%)	15 (9)	2 (12)	0.72	
Previous pregnancies; *n* (%)	53 (32)	10 (59)	**0.03**	
Previous children; *n* (%)	11 (7)	0 (0)	0.27	
**Started IVF treatments**				
Total FSH/hMG dose (IU); median (IQR)	1800 (950)	1975 (1100)	0.20	
Duration of COS (days); median (IQR)	10 (2)	10 (3)	0.82	
Type of COS protocol used; *n* (%)			1.00	
Agonist	1 (0.4)	0 (0)		
Antagonist	223 (99.6)	22 (100)		
Number of retrieved oocytes; median (IQR)	8 (8)	6 (8)	0.14	
Type of fertilization			0.82	
Conventional IVF	130 (58)	12 (55)		
ICSI	93 (42)	10 (45)		
Reasons for cycle cancellation; *n* (%)			1.00	
Total freeze due to OHSS	78 (35)	6 (27)		
No retrieved oocytes	1 (0.4)	0 (0)		
Total fertilization failure	11 (5)	0 (0)		
No blastocyst development	7 (3)	0 (0)		
Severe cleavage stage embryo fragmentation	1 (0.4)	0 (0)		
Nr. of blastocysts; median (IQR)	2 (5)	2 (3)	0.48	0.43^†^
Nr. of cryopreserved blastocysts; median (IQR)	1 (4)	1 (3)	0.43	0.33^†^
Fertilization rate (%); median (IQR)	70 (61)	71 (47)	0.45	0.07^†^
Blastocyst formation rate (%); median (IQR)	33 (61)	22 (52)	0.47	0.96^†^
Blastocyst formation; *n* (%)	158 (71)	14 (64)	0.48	0.52^††^

*HPV*, human papillomavirus; *BMI*, body mass index; *IQR*, interquartile range; *FSH/hMG*, follicle-stimulating hormone/human menopausal gonadotropin; *COS*, controlled ovarian stimulation; *ICSI*, intracytoplasmic sperm injection; *OHSS*, ovarian hyperstimulation syndrome. Adjusted *p*-value: statistical comparison between HPV-negative and HPV-positive groups. Adjustments were made for the number of retrieved oocytes, total amount of progressively motile sperm after preparation, duration of infertility, male and female ages, sperm concentration after preparation, and the fertilization method used. Linear regression models (†) were applied for continuous outcomes and logistic regression (††) was used for binary outcomes

The VIM analysis from the optimal XGBoost model, detailed in Supplementary Table 3, indicated that HPV presence in semen did not contribute to model accuracy, as it was not selected for any splits. The “number of retrieved oocytes” emerged as the most influential factor (gain 10.80), followed by “total amount of progressive sperm after preparation” and “snuffing status of the male,” with gain values of 3.71 and 2.54, respectively. Attempts to evaluate the predictive capabilities of the model for fertilization and blastocyst formation rates did not yield robust results for further VIM analysis.

## Discussion

This study explored HPV prevalence and its association with seminal parameters, identifying a point prevalence of HPV of 8.9% in semen samples. HPV-positive men showed a trend toward higher median total sperm motility compared to HPV-negative men. No significant differences were observed in other semen parameters between the groups. However, in the non-male infertility subgroup, HPV-positive men had significantly higher total sperm motility and progressive motile sperm compared to HPV-negative men. In the unexplained infertility subgroup, HPV-positive men demonstrated higher progressive motility, with total sperm motility approaching statistical significance. Notably, the presence of HPV in semen did not significantly affect IVF outcomes under the conditions examined, suggesting a limited impact on reproductive success in this cohort.

Among men undergoing IVF treatment in this study, the prevalence of HPV in semen samples was lower than previously reported in the literature. A systematic review by Yang et al. documented an HPV prevalence of 17.4% among men with idiopathic infertility [43]. Similarly, Kato et al. reported a prevalence of 12.5% among infertile Japanese men [44]. Two meta-analyses further demonstrated a significantly higher HPV prevalence in semen from infertile couples compared to the general population, with a prevalence of 20.9 [21] and 20.4% among fertility clinic attendees [45].

The relationship between HPV infection and seminal parameters has been a topic of considerable interest in reproductive medicine. Our findings showed no statistically significant differences in clinical and semen parameters between HPV-positive and HPV-negative men. This aligns with several studies that have reported similar findings regarding the lack of significant differences in sperm concentration, motility, and semen volume between these groups [46–48]. However, our results also indicate a trend toward higher total sperm motility in HPV-positive men, particularly within the non-male infertility subgroup, where significant differences in motility measures were observed. This unexpected finding is consistent with the study by Notari et al. [49], which reported enhanced motility in HPV-positive spermatozoa. Similarly, studies conducted by Connelly et al. [50] and Brossfield et al. [51], which investigated the effects of HPV-DNA exposure on washed sperm cells, suggested that specific conditions associated with HPV infection may lead to improved sperm motility. HPV may bind to spermatozoa through aquaporins, as reported by Pellavio et al. [52]. Different HPV genotypes can attach to various regions of the sperm cell, such as the head, midpiece, or other segments, potentially interacting with distinct surface receptors, as described by Schillaci et al. [53], Foresta et al. [54], and Kato et al. [44]. This interaction between HPV and sperm membrane receptors could trigger intracellular signaling, potentially altering functions of aquaporins and/or inducing Ca^2^⁺ release, which may enhance rapid progressive sperm motility. Nevertheless, these observations contrast with many previous studies, which have consistently reported impaired sperm motility in HPV-positive men [16, 44, 55–58]. The discrepancies in findings across studies may be attributed to variations in study populations, methodological approaches, and the specific HPV genotypes examined. Further research is warranted to clarify the potential influence of HPV on sperm motility and its mechanism, accounting for these methodological differences and possible confounding factors.

In the present study, HPV 51 emerged as the most frequently detected HR HPV genotype in semen, a finding that aligns with previous studies identifying HPV 16 and 51 as commonly present in semen samples from infertile men [21, 59]. However, our observation of a low prevalence of HPV 16 contrasts with prior research, which has reported higher detection rates of this genotype among HPV-positive men [45, 48]. This discrepancy is particularly notable in comparison to a previous study on HPV prevalence in the Swedish population, which identified HPV 16 as the most dominant HR HPV genotype in both men and women [60, 61]. Notably, HPV 51 is not included in the HPV vaccines currently used in Sweden, namely Gardasil® and Gardasil 9®, which are administered through the school-based vaccination program [62]. This exclusion may explain the predominance of HPV 51 as the most frequently detected HR HPV genotype in our study population.

Machine learning techniques have increasingly been applied in reproductive medicine to enhance the predictive accuracy of embryological outcomes by identifying complex patterns within reproductive data with minimal human intervention [63]. In the present study, the XGBoost model identified the “number of retrieved oocytes” as the most significant predictor of successful blastocyst formation, consistent with existing evidence highlighting the role of oocyte availability in fertility treatment success [63–65]. Additional significant predictors included the “total amount of progressive sperm after preparation” and “snuffing status of the male,” underscoring the importance of sperm quality and lifestyle factors in reproductive outcomes [63]. Notably, HPV presence in semen was not associated with fertilization rates or blastocyst formation, aligning with previous studies that found no correlation between seminal HPV status and IVF success rates [47, 48, 66, 67]. The ML analysis further reinforced this finding, as HPV status was not selected for any decision splits in the XGBoost model, while traditional clinical and semen parameters remained the primary predictors of embryological outcomes. These results clearly indicate that oocyte quantity and sperm motility, rather than HPV presence, may play a more decisive role in determining IVF success, consistent with established literature [65, 68].

The impact of seminal HPV on IVF outcomes has been widely investigated with mixed findings. While some studies suggest that HPV presence in semen may be associated with reduced sperm quality, these effects do not consistently affect IVF success rates. Souho et al. noted that sperm cells can adsorb HPV particles in the equatorial region of the head without impairing their ability to fertilize an oocyte, though the impact on later stages of fertilization and embryo development remains uncertain [69]. Moreover, studies by Muscianisi et al. and Wang et al. found no significant association between HPV infection in semen and key reproductive parameters such as sperm count and motility, further supporting the notion that seminal HPV presence may not be a critical determinant of IVF outcomes [14, 70].

The primary strength of this study lies in the application of ML techniques to identify predictors of IVF success, marking a significant advancement in reproductive medicine. This approach enables the analysis of complex, multifactorial data with minimal human bias, offering a more comprehensive understanding of the interactions between male fertility factors and IVF outcomes. The XGBoost model, known for its ability to manage large datasets and deliver high predictive accuracy, strengthens traditional statistical methodologies by identifying key determinants of reproductive success [71]. Notably, the analysis revealed that conventional factors, such as the number of retrieved oocytes and total progressive sperm after preparation, were the most influential predictors of blastocyst formation. Importantly, the presence of HPV in semen did not significantly affect the model’s predictive performance, challenging assumptions of a universal detrimental impact of HPV on male fertility [19, 72]. These findings emphasize the relevance of established clinical parameters as stronger predictors than HPV status and encourage further investigation into the mechanisms of sperm-HPV interactions and the role of male fertility factors in IVF success [49].

Several limitations should be considered when interpreting the findings of this study. Although the sample size was relatively substantial, as it exceeded the minimum required for a reliable estimation of HPV prevalence in semen, it may still be insufficient to draw definitive conclusions for the broader population, especially given the variability in HPV prevalence across geographic and demographic groups [73]. A notable limitation of this study is the restricted number of detectable HPV genotypes, as the applied technique could identify only 14 genotypes. This limitation may have led to an underestimation of HPV prevalence in semen. Future studies employing broader genotyping panels or next-generation sequencing technologies may provide a more comprehensive understanding of the relationship between HPV infection and male fertility outcomes. Furthermore, the study was not specifically designed or powered to assess reproductive outcomes such as live birth and miscarriage rates, leading to the exclusion of these results from the present analysis. Another critical limitation is the absence of postpreparation HPV analysis, as standard sperm preparation techniques have been shown to be ineffective in fully eliminating HPV DNA from semen, raising questions about its potential influence on IVF outcomes [74]. Future research should aim to assess the relationship between seminal HPV status and long-term reproductive outcomes, such as clinical pregnancy, live birth, and miscarriage rates. Moreover, testing the efficacy of advanced sperm preparation methods—beyond conventional density gradient centrifugation—in reducing HPV load in semen may offer further insights into minimizing potential virological impacts on fertilization and embryo development.

Although the use of ML techniques provides valuable insights, this approach has inherent limitations. The model’s performance relies heavily on data quality and completeness, as potential biases in the dataset can affect predictive accuracy [75]. However, it is important to acknowledge that robust ML models require large, multicenter datasets with standardized data collection to generate significant results. In this study, data were obtained from a single center, which limits the generalizability of the ML-driven analysis. Therefore, while ML was applied as an exploratory tool, further studies with larger datasets are needed to validate its predictive power. Despite these limitations, this study was conducted using high-quality data, which is likely to contribute to reliable results. Additionally, ML models can be challenging to interpret, complicating the determination of the biological relevance of the identified predictors in the context of male fertility [46].

## Conclusion

This study found a low prevalence of HPV in semen samples from men undergoing IVF treatment in Sweden, with HR genotypes being more prevalent. Elevated sperm motility in HPV-positive men was observed in certain subgroups, challenging the established notion of HPV’s detrimental effect on sperm motility as suggested by the literature. However, no significant impact on embryological outcomes, such as fertilization or blastocyst formation, was observed. These findings suggest that routine HPV screening in semen may not be clinically necessary in the context of IVF planning, as HPV presence did not significantly impact embryological outcomes in this cohort.

## Supplementary Information

Below is the link to the electronic supplementary material.

Supplementary file1 (DOCX 17 KB)

Supplementary file2 (DOCX 18 KB)

Supplementary file3 (DOCX 16 KB)

## Data Availability

The datasets generated and analyzed during the current study are not stored in a public repository. However, the data are available upon request from the corresponding author. Interested parties can contact the corresponding author directly for access to the relevant data.

## Code availability

The datasets generated and analyzed during the current study are not stored in a public repository. However, the data are available upon request from the corresponding author. Interested parties can contact the corresponding author directly for access to the relevant data.
