# Supplementary material for: Semen HPV and IVF: insights from infection prevalence to embryologic outcomes
Source: J Assist Reprod Genet. 2025 May 22;42(6):2053–66. doi: 10.1007/s10815-025-03513-6 (PMC12226446; doi:10.1007/s10815-025-03513-6)
Supplement: Supplementary file 1 — Supplementary file1 (DOCX 17 KB) [file 10815_2025_3513_MOESM1_ESM.docx]

**Supplementary Tab. 1** Independent variables used in the IVF outcome forecasting models.

| **Category** | **Feature** |
| --- | --- |
| **Demographic factors** |  |
|  | Age (male and female) |
|  | BMI (male and female) |
|  | Smoking/snuffing status (male and female) |
|  | Previous children (male and female) |
|  | Previous pregnancies (female) |
|  | Previous diseases (male and female) |
| **IVF-related factors** |  |
|  | Indication for IVF |
|  | Infertility duration |
|  | IVF protocol |
|  | Total amount of gonadotropins used |
|  | Duration of IVF stimulation |
|  | Number of retrieved oocytes |
|  | Fertilization method used |
| **Sperm analysis**  **(pre-preparation)** |  |
|  | Sperm concentration |
|  | Total sperm count |
|  | Total sperm motility |
|  | Progressive sperm |
|  | Non-progressive sperm |
|  | Motility score |
|  | Semen volume |
|  | Infection within one week from sperm analysis |
| **Sperm analysis**  **(post-preparation)** |  |
|  | Semen volume used for preparation |
|  | Semen volume after preparation |
|  | Sperm concentration after preparation |
|  | Total sperm count after preparation |
|  | Concentration of progressive sperm after preparation |
|  | Progressive sperm after preparation |
|  | Motility score after preparation |
|  | Total amount of progressive sperm after preparation |
| **Semen HPV status** |  |
|  | Presence of semen HPV |
|  | Presence of low-risk semen HPV |
|  | Presence of high-risk semen HPV |
