# Supplementary material for: Semen HPV and IVF: insights from infection prevalence to embryologic outcomes
Source: J Assist Reprod Genet. 2025 May 22;42(6):2053–66. doi: 10.1007/s10815-025-03513-6 (PMC12226446; doi:10.1007/s10815-025-03513-6)
Supplement: Supplementary file 2 — Supplementary file2 (DOCX 18 KB) [file 10815_2025_3513_MOESM2_ESM.docx]

**Supplementary Tab. 2** Tested and optimal hyperparameters used for model training. The table presents the range of tested values and the final selected (optimal) values for each hyperparameter tuned during the training process.

| **Model** | **Hyperparameter** | **Values Tested** | **Optimal Value** |
| --- | --- | --- | --- |
| **Random Forest** | Number of trees | 20, 50, 100, 200, 500, 1000, 2000, 5000 | 100 |
|  | Maximum tree depth | 3, 5, 15, None | 15 |
|  | Splitting criterion | gini | gini |
| **Decision Tree** | Maximum tree depth | 1-10, 15, 20, 30, None | 2 |
|  | Minimum samples per leaf | 1, 100, 1 | 22 |
|  | Splitting criterion | gini | gini |
| **SVM (Linear)** | Regularization parameter (C) | 0.01 to 10 (step=0.01) | 0.3 |
| **SVM (RBF)** | Regularization parameter (C) | 0.01 to 10 (step=0.01) | 4.82 |
|  | Kernel coefficient (gamma) | 0.01 to 1 (step=0.01) | 0.03 |
| **SVM (Polynomial)** | Regularization parameter (C) | 0.01 to 10 (step=0.01) | 0.01 |
|  | Polynomial degree | 2, 3, 4, 5 | 5 |
|  | Kernel coefficient (gamma) | 1 | 1 |
| **XGBoost** | Number of trees | 20, 50, 100, 200, 500, 1000, 2000, 5000 | 200 |
|  | Maximum tree depth | 3, 5, 15, None | 3 |
|  | Learning rate | 0.01, 0.1 | 0.01 |
|  | Subsample ratio (training instances) | 0.5, 0.6, 0.7, 0.8, 0.9, 1.0 | 0.9 |
|  | Column subsample ratio (colsample_bytree) | 0.5, 0.6, 0.7, 0.8, 0.9, 1.0 | 1.0 |
| **Elastic-Net** | L1 ratio (Elastic-Net mixing parameter) | 0.1 to 1 (step=0.1) | 0.1 |
|  | Maximum number of iterations | 10000 | 10000 |
