# Supplementary material for: Semen HPV and IVF: insights from infection prevalence to embryologic outcomes
Source: J Assist Reprod Genet. 2025 May 22;42(6):2053–66. doi: 10.1007/s10815-025-03513-6 (PMC12226446; doi:10.1007/s10815-025-03513-6)
Supplement: Supplementary file 3 — Supplementary file3 (DOCX 16 KB) [file 10815_2025_3513_MOESM3_ESM.docx]

**Supplementary Tab. 3** Variable Importance Measure (VIM) based on Extreme Gradient Boosting (XGBoost) Gain for assessing factors influencing embryological outcomes.

| **Feature name** | **Gain value** |
| --- | --- |
| Number of retrieved oocytes | 10.80 |
| Total amount of progressive sperm after preparation | 3.71 |
| Snuffing status (male) | 2.54 |
| Total amount of gonadotropins used | 2.54 |
| Motility score after preparation | 2.19 |
| Total sperm motility | 2.16 |
| Semen volume | 2.07 |
| Infertility duration | 2.05 |
| Age (male) | 1.99 |
| BMI (female) | 1.97 |
| Duration of IVF stimulation | 1.88 |
| Sperm concentration after preparation | 1.79 |
| Concentration of progressive sperm after preparation | 1.73 |
| Semen volume used for preparation | 1.62 |
| Total sperm count after preparation | 1.62 |
| Sperm concentration | 1.53 |
| Age (female) | 1.51 |
| BMI (male) | 1.40 |
| Progressive sperm after preparation | 1.34 |
| Motility score | 1.31 |
| Progressive sperm | 1.27 |
| Previous diseases (female) | 1.18 |
| Total sperm count | 1.00 |
| Indication for IVF | 0.85 |
